# Experimental Evidence of *ω*-3 Polyunsaturated Fatty Acid Modulation of Inflammatory Cytokines and Bioactive Lipid Mediators: Their Potential Role in Inflammatory, Neurodegenerative, and Neoplastic Diseases

**DOI:** 10.1155/2013/743171

**Published:** 2013-04-17

**Authors:** Gabriella Calviello, Hui-Min Su, Karsten H. Weylandt, Elena Fasano, Simona Serini, Achille Cittadini

**Affiliations:** ^1^Institute of General Pathology, School of Medicine, Catholic University, 00168 Rome, Italy; ^2^Department of Physiology, National Taiwan University College of Medicine, Taipei 100, Taiwan; ^3^Department of Gastroenterology, Hepatology and Endocrinology and Experimental and Clinical Research Center (ECRC), Charité University Medicine, 13353 Berlin, Germany

## Abstract

A large body of evidence has emerged over the past years to show the critical role played by inflammation in the pathogenesis of several diseases including some cardiovascular, neoplastic, and neurodegenerative diseases, previously not considered inflammation-related. The anti-inflammatory action of *ω*-3 polyunsaturated fatty acids (PUFAs), as well as their potential healthy effects against the development and progression of the same diseases, has been widely studied by our and others' laboratories. As a result, a rethinking is taking place on the possible mechanisms underlying the beneficial effects of *ω*-3 PUFAs against these disorders, and, in particular, on the influence that they may exert on the molecular pathways involved in inflammatory process, including the production of inflammatory cytokines and lipid mediators active in the resolving phase of inflammation. In the present review we will summarize and discuss the current knowledge regarding the modulating effects of *ω*-3 PUFAs on the production of inflammatory cytokines and proresolving or protective lipid mediators in the context of inflammatory, metabolic, neurodegenerative, and neoplastic diseases.

## 1. Introduction 

A number of studies has supported the hypothesis that a wide range of chronic diseases, including cardiovascular [1, 2], neoplastic [3, 4], and neurodegenerative diseases [5, 6], may be beneficially influenced by the increased intake of dietary *ω*-3 polyunsaturated fatty acids (PUFAs). Many molecular mechanisms have been invoked to explain such a pleiotropic effect of these fatty acids (FAs) [3, 7]. Meanwhile, plenty of experimental preclinical data has suggested the potential healthy effects of these compounds also on diseases with a clear inflammatory/immune pathogenesis, including asthma, rheumatoid arthritis, or inflammatory bowel diseases [8, 9].

It should be noted that conflicting results have been obtained so far by the clinical trials conducted in this research field [2, 8–12]. However, on the whole, the human intervention studies [9, 10, 12] are still too scarce in number and often show severe pitfalls. The *ω*-3 PUFA potential of modulating the molecular pathways involved in the inflammatory processes has been intensely investigated in experimental preclinical studies, and a substantial body of evidence has accumulated over the years to clarify that an inflammatory component may play an essential role in the pathogenesis of diseases that before had not been considered inflammation-related, such as some cardiovascular, neoplastic, and neurodegenerative diseases, and particularly metabolic disease, inflammation-associated cancers, Alzheimer's disease (AD), cognitive decline of aging, and so forth [13–16]. The altered production of inflammatory cytokines and bioactive lipid mediators with proresolving or protecting activities, either by inflammatory cells or by cells residing in the affected tissues, has attracted considerable attention as possible critical steps in the pathogenesis of these diseases. 

On this basis, in the present review we will analyze the published evidence that has so far contributed to show the relationships existing between the potential beneficial effects of *ω*-3 PUFAs against several inflammatory diseases, as well as against pathologies having an inflammatory component, and the modulatory effects that these FAs exert on the amount and quality of inflammatory cytokines and bioactive lipid mediators produced during the development and progression of these diseases. In the first part we will treat in general the anti-inflammatory effects of these FAs, with particular attention to important inflammatory and dysmetabolic conditions affecting different tissues and organs, trying to elucidate the underlying mechanisms and reviewing the modulating effects that these FAs may have on cytokine and lipid mediators productions in these disorders. Afterwards, we will examine in detail the relationships between the anti-inflammatory potential of these FAs and their beneficial effects against the development and progression of neurodegenerative diseases and cancer.

## 2. *ω*-3 PUFA Chemistry and Biochemistry

**Figure 1:**
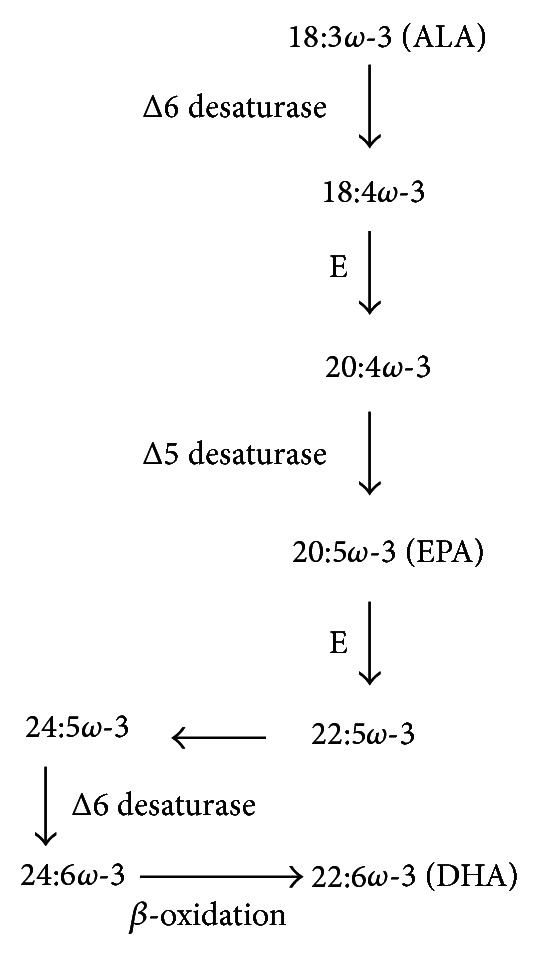
Endogenous synthesis of long-chain-PUFAs. ALA: *α*-linolenic acid; DHA: docosahexaenoic acid; EPA: eicosapentaenoic acid; E: elongase. 24:5*ω*-3 is converted to DHA by *β*-oxidation in peroxisomes.

There is wide consensus that among the *ω*-3 PUFAs the most powerful are the *ω*-3 long-chain-PUFAs (*ω*-3 LCPUFAs) docosahexaenoic (DHA, 22:6*ω*-3) and eicosapentaenoic acid (EPA, 20:5*ω*-3) [17]. We can produce them endogenously metabolically converting *α*-linolenic acid (ALA, 18:3*ω*-3), an essential PUFA largely available in vegetables, through sequential desaturations and elongations followed by peroxisomal *β*-oxidation [18] (Figure 1). However, the efficiency of these conversions is quite low in the presence of high levels of linoleic acid (LA, 18:2*ω*-6), as those present in many Western diets [19]. Instead, we can directly obtain EPA and DHA by components of our diet that contain high levels of these FAs, such as fish and seafood.

**Figure 2:**
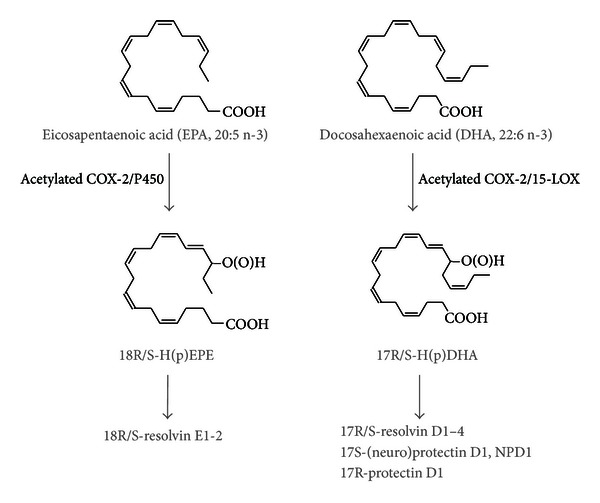
Production of anti-inflammatory lipid mediators from EPA and DHA. E-series resolvins are formed via a common precursor 18R/S-H(p)EPE, while D-series resolvins are synthesized via the common precursor 17R/S-H(p)DHA. Note that anti-inflammatory activity has been described for several of the R, as well as the S stereoisomers.

Irrespective of their dietary origin, *ω*-3 PUFAs, as well as *ω*-6 PUFAs can be converted in our cells and tissues into potent lipid mediators playing an important role in the regulation of inflammation. For many years the predominant focus in the understanding of lipid mediators was on the *ω*-6 arachidonic acid (AA) and its derived compounds such as prostaglandins (PG) and leukotrienes (LT), which were initially described early in the 20th century. Generally, these *ω*-6 PUFA-derived lipid mediators promote inflammation whereas a wide variety of experimental studies has assigned *ω*-3 PUFAs as anti-inflammatory. This was traditionally attributed to their ability to inhibit the formation of *ω*-6 PUFA-derived proinflammatory PG and LT. However, newly discovered hydroxylated lipid metabolites derived from EPA and DHA, such as resolvins and protectins (Figure 2), are potent anti-inflammatory lipid mediators derived directly from *ω*-3 PUFAs with distinct pathways of action [20–23].

Most of preclinical *in vitro* and *in vivo* studies have been conducted treating cells and animals with fish or algal oils containing EPA and DHA, or with purified EPA or DHA. Moreover, a recently developed transgenic mouse model, the *fat-1* mouse [24], that has endogenously increased *ω*-3 PUFAs has been used in a large number of studies. This transgenic mouse expresses a *Caenorhabditis elegans* desaturase, leading to the formation of endogenously high levels of *ω*-3 PUFAs from *ω*-6 PUFAs. This model has been extremely useful to establish the effect of increased *ω*-3 PUFAs on several chronic pathologies, since it offers several advantages over conventional dietary approaches using *ω*-3 PUFAs, as it eliminates confounding factors of diet (content of, e.g., antioxidants) that could have significant effects themselves on animal models of inflammatory disease. 

## 3. Effects of *ω*-3 PUFAs in Disorders of GI-Tract and in Metabolic Diseases: Involvement of Cytokines and Lipid Mediators

### 3.1. Effect of *ω*-3 PUFAs in the Colon

Using this transgenic mouse model in experimental dextran sodium sulfate (DSS) colitis demonstrated protection from colitis in the *fat-1* mice with increased *ω*-3 PUFA status. This was accompanied by decreased nuclear factor-*κ*B (NF-*κ*B) activity and decreased expression of tumor necrosis factor-*α* (TNF-*α*), inducible nitric oxide synthase (NOS), and interleukin-1*β* (IL-1*β*). Analyzing hydroxylated lipid mediators derived from *ω*-3 PUFAs in this context demonstrated significant formation of anti-inflammatory resolvins [25]. Another study with DSS colitis in *fat-1* mice established decreased cyclooxygenase-2 (COX-2) expression and decreased PGE_2_ levels in the *fat-1* mice and confirmed the suppression of the cytokine response in these mice with a lower expression of IL-18, IL-1*α*, IL-1*β*, IL-6, monocyte chemotactic protein-1,2,3 (MCP-1,2,3), matrix metalloproteinase-9 (MMP-9), and TNF-*α* [26]. However, when we compare these findings obtained in animals to studies in humans, the picture becomes less clear. While one study has shown beneficial effects of *ω*-3 PUFAs on human Crohn's disease [27], the data so far are not conclusive, as another trial in patients with Crohn's disease in remission could not find a benefit of supplementation with *ω*-3 PUFAs [28]. Furthermore, a Cochrane Database systematic review could not establish a benefit for *ω*-3 PUFA treatment for the maintenance of remission in ulcerative colitis [29]. 

### 3.2. Effect of *ω*-3 PUFAs on the Liver

In *fat-1* mice with a balanced *ω*-6/*ω*-3 PUFA tissue content, the increased *ω*-3 PUFA content decreased the inflammatory response in the macrophage-dependent D-Gal/LPS acute hepatitis model. This was associated with decreased plasma TNF-*α* levels and reduced expression of IL-1*β*, interferon-*γ* (IFN-*γ*) and IL-6 in *fat-1* mice, leading to a decreased rate of apoptosis in livers from *fat-1* animals [30]. This could be due to the formation of *ω*-3 PUFA-derived lipid mediators, as lipidomics analyses of liver tissue revealed significantly increased levels of the *ω*-3 PUFA-derived 18-hydroxyeicosapentaenoic acid (18-HEPE) and 17-hydroxydocosahexaenoic acid (17-HDHA) in the livers of *fat-1* animals with chemically induced liver tumors [31]. In another study, DHA supplementation led to increased formation of DHA-derived lipid mediators such as 17-HDHA and protectin D1, which were able to protect the liver from CCL4-induced inflammatory damage [32]. The study also showed that 17-HDHA can suppress TNF-*α* secretion from cultured murine macrophages. This was confirmed in other *in vitro* experiments showing that 17-HDHA—and EPA-derived 18-HEPE—could effectively suppress LPS-triggered TNF-*α* formation in a murine macrophage cell line [31]. 

### 3.3. Metabolic Disease and *ω*-3 PUFAs

Other evidence demonstrates that *ω*-3 PUFAs may also play an important role in the alleviation of metabolic disturbances in the liver. A study using a high-fat diet in* fat-1* and wild-type mice demonstrated very mild steatosis in *fat-1* mice as compared to a moderate-to-severe steatosis in wild-type animals with normal transaminase levels in *fat-1* mice as compared to elevated values in high-fat diet-fed wild-type mice. This study also demonstrated the well-established lipidologic effects of *ω*-3 FAs with significantly decreased serum triglycerides, very low-density lipoprotein cholesterol, and chylomicrons on *fat-1* mice [33]. These findings are in accordance with other data using dietary supplementation of *ω*-3 PUFAs in which these FAs were shown to alleviate hepatic steatosis and to up regulate PPAR*γ*, glucose transporter-2 (GLUT-2) and GLUT-4, and insulin receptor signaling (IRS-1/IRS-2) genes as well as the anti-inflammatory and insulin-sensitizing adiponectin, while concomitantly inducing AMP-activated protein kinase (AMPK) phosphorylation. Omega-3-PUFA suppressed the formation of *ω*-6-PUFA-derived eicosanoids and triggered formation of *ω*-3-PUFA-derived lipid mediators [34]. Interestingly, the administration of *ω*-3 PUFA-rich parenteral nutrition has been established as beneficial in pediatric patients with parenteral nutrition-associated liver disease. In these children administration of a fish-oil-based parenteral nutrition led to a significant decrease of the serum bilirubin as well as of triglycerides, and total and LDL-cholesterol [35]. These metabolic effects of *ω*-3 PUFAs leading to the alleviation of liver steatosis, as well as lipid and glucose status are therefore associated with the clinically proven—and widely used—beneficially effect of *ω*-3 PUFAs in hypertriglyceridemia. Hypertriglyceridemia is one of the indications for which high-dose *ω*-3 PUFA preparations are approved by the Food and Drug Administration (FDA) and the European regulatory authorities, and many studies consistently show this effect, with doses of the *ω*-3 PUFA preparations of up to 12 g/d [36–38]. Furthermore, as high triglyceride levels predispose to the development of pancreatitis [39, 40], it is noteworthy that experimental data suggest an anti-inflammatory effect of *ω*-3 PUFAs also in the context of pancreatitis. In acute pancreatitis, *fat-1* mice showed a decreased systemic inflammatory response as measured by plasma IL-6 levels and neutrophil infiltration in the lung, as well as a trend towards decreased pancreatic necrosis. Possibly of importance for the prevention of long-term complications of chronic pancreatitis, such as chronic pain and exocrine and endocrine pancreatic insufficiency, chronic pancreatitis in *fat-1* was associated with decreased pancreatic fibrosis [41]. Among others, these animal data and some human studies [42, 43] therefore suggest a beneficial potential for *ω*-3 PUFA supplementation in pancreatitis.

### 3.4. Lipidomics in Metabolic Liver Disease

Recent studies have analyzed lipidomic aspects in the context of nonalcoholic steatohepatitis (NASH) pathology [44, 45], demonstrating an increase in the ratio of *ω*-6 to *ω*-3 PUFAs in NASH liver tissue as well as in the plasma levels of the AA metabolites 5-HETE, 8-HETE, and 15-HETE in the progression from normal to NASH. These data indicate that the liver is critical not only for lipoprotein and triglyceride metabolism, but also for the conversion of essential PUFAs to bioactive lipid mediators, due to the expression of COX, lipoxygenase (LOX), and cytochrome P450 enzymes. The *ω*-6/*ω*-3 PUFA ratio in liver tissue therefore probably determines the lipid mediator profile generated and could thus be an important factor in the development of metabolic (liver) disease. Mass spectrometry techniques allowing for a comprehensive and concomitant assessment of a multitude of *ω*-3 and *ω*-6 PUFAs and their derived lipid metabolites in human blood and tissue samples [46] will thus have an important role now for the evaluation of lipid metabolite formation and of their role in different physiological and pathophysiological contexts. Measurement of *ω*-3 derived lipid metabolites and mediators such as 18-HEPE, 17-HDHA, and their resolvins in blood might thus be one of the most important tools to better understand the biological impact of *ω*-3 PUFAs on the human body (Figure 2).

## 4. Beneficial Effects of *ω*-3 PUFAs on Neurodegenerative Diseases: Involvement of Cytokines and Lipid Mediators

Among *ω*-3 PUFAs, DHA is specifically enriched in the brain and is essential for normal neurological function [6]. In the mammalian brain, lipids make up 10% of the fresh weight and 50% of the dry weight, and DHA and AA (20:4*ω*-6) are the major PUFAs found in the neuronal membrane [47]. Most DHA and AA accumulation in the brain takes place during brain development in the perinatal period from the beginning of the third trimester of gestation to 2 years after birth in humans and from prenatal day 7 to postnatal day 21 in rats [48–50]. However, hippocampal DHA levels decrease with age in rats [51, 52] and are reduced in the neurodegenerative Alzheimer's disease (AD) [22, 53, 54]. Deficiency of hippocampal DHA is associated with reduced learning and memory ability in rats [55] and with cognitive decline in AD patients [56]. 

Although the liver is the major site of DHA biosynthesis [57], DHA can be synthesized locally in the developing brain [58, 59]. In contrast, the human adult can convert ^13^C-labeled ALA to EPA and, to a lesser extent, docosapentaenoic acid (22:5*ω*-3), but very little is converted to DHA [60]. In addition, human blood levels of EPA, but not DHA, are increased in healthy humans supplemented with ALA (2–40 g/day for 3–26 weeks) or EPA (1–4 g/day for 4–12 weeks) [61]. The 15-lipoxygenase-1 expression and NPD1 levels are reduced in the hippocampus of AD patients [22]. These findings indicate that the developing brain has the ability to synthesize and take up DHA, while, in the adult, DHA synthesis is low, and, in the patient of AD, NPD1 biosynthesis is reduced. 

### 4.1. Specific Effect of *ω*-3 PUFAs on Neuron Protection

Evidence is accumulating that neuron protection is provided by *ω*-3 fatty acids, but not other fatty acids. DHA or NPD1, but not AA, attenuates TNF-*α*- and H_2_O_2_ -induced apoptosis of human retina pigment epithelial ARPE-19 cells [62]. DHA, but not 22:5*ω*-6, AA, or oleic acid (OA, 18:1*ω*-9), protected mouse neuroblastoma neuro 2A cells against apoptosis induced by 2-day serum starvation [63, 64], and DHA, but not AA or OA, prevented oxidative stress-induced apoptosis of neuro 2A cells [65]. DHA, but not AA, OA, or palmitic acid (16 : 0), prevented apoptosis of rat retina photoreceptor cells during development or induced by oxidative stress [66, 67]. ALA, but not 16 : 0, provided protection against ischemia-induced hippocampal cell death and prevented kainic acid-induced seizures in rats [68]. These findings indicate a specific effect of *ω*-3 PUFAs in neuron protection.

### 4.2. *ω*-3 PUFAs Reverse Age-Related Inflammation Changes

Levels of mRNAs coding for major histocompatibility complex molecule II and CD40, markers of microglial cell activation indicating neuronal inflammation, and protein levels of IFN-*γ* and IL-1*β* were increased in the hippocampus in aged rats (22-month-old) compared to young rats (4-month-old), and these effects on aged rats were overcome by supplementation with EPA (125 mg/kg/day for 4 weeks) [69]. 

### 4.3. *ω*-3 PUFAs in AD Prevention

AD is a progressive neurodegenerative disease characterized by dementia with impaired cognitive performance and accounts for 70% of dementia patients. The main pathology of AD is related to extracellular deposits of diffusible assemblies (oligomers) of amyloid *β* peptide (A*β*), fibrillar aggregated of A*β* as plaques, and of intracellular phosphorylated tau protein as tangles, which cause neuronal death [70–72]. In AD patients, the hippocampus is one of the first brain regions to suffer damage [73, 74]. In these patients, DHA and Neuroprotectin D1 (NPD1) levels are reduced in the hippocampus but are unchanged in the frontal cortex, thalamus, or occipital lobes [22, 49]. Treatment of human SH-S5Y5 neuronal cells with DHA inhibits the formation of A*β* fibrills and oligomers and their cytotoxicity [75]. Moreover, in studies on a primary coculture of human neurons and glia supplemented with DHA, NPD1 biosynthesis is increased, A*β* production is reduced, antiapoptotic gene expressions, Bcl-2 and Bfl-1, are upregulated, and cell survival is increased [22].

In addition, NPD1 downregulates A*β*-induced expression of proinflammatory elements including COX-2, tumor necrosis factor alpha (TNF-*α*), and a TNF-*α*-inducible pro-inflammatory element B-94 to promote cell survival in the human neuronal-glial cells [76]. Moreover, DHA enhances NPD1 synthesis and NPD1 reduces IL-1*β*-stimulated COX-2 expression, and DHA/NPD1 up regulates anti-apoptotic Bcl-2 and Bfl-1 proteins and down-regulate proapoptotic BID, BAX, BAD, and caspase-3 proteins in human ARPE-19 cells [62, 77]. These studies suggest that DHA increases NPD1 biosynthesis, reduces A*β* production, and inhibits inflammatory cytokine secretion in neuron cells.

In studies of *ω*-3 PUFA supplementation in an AD animal model, A*β* plaques in the hippocampus were reduced in aged (22.5-month-old) AD mice fed with a DHA-enriched diet (0.6% w/w in chow diet) for about 103 days [78], DHA levels were increased, soluble A*β* levels reduced, and levels of phosphorylated tau protein decreased in the brain in adult (3-month-old) AD mice fed with a DHA-enriched diet (1.3% w/w in control diet) for 3–9 months [79] and reactive oxygen species levels and the number of apoptotic neurons in the hippocampus were decreased, hippocampal DHA levels increased, and radial-maze learning memory performance improved in A*β*-infused adult rats supplemented with DHA or 20:5*ω*-3 (300 mg/kg/day for 12 weeks) [80–82]. Phosphorylation of the antiapoptotic protein BAD was increased and levels of oxidized proteins decreased in the cortex and water-maze learning memory performance improved in aged (17-month-old) AD mice fed with an *ω*-3 fatty acid-deficient diet when the diet was supplemented for 103 days with DHA (0.6% w/w in the *ω*-3 deficient diet) [83]. These findings indicate that reduced brain DHA levels are restored, NPD1 biosynthesis is increased, A*β* production is reduced, antiapoptosis proteins are increased, and learning memory improved, by *ω*-3 PUFAs consumption.

**Figure 3:**
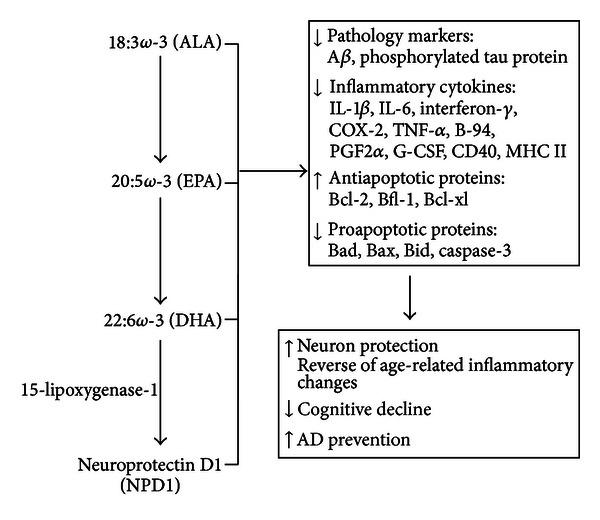
Preventive effect of *ω*-3 PUFAs on the neurodegenerative Alzheimer's disease. ALA: *α*-linolenic acid; DHA docosahexaenoic acid; EPA: eicosapentaenoic acid; DHA: is converted to NPD1 by 15-lipoxygenase-1 in neuronal cells. *ω*-3 PUFAs reduce AD pathology, inhibit inflammatory cytokines secretion, upregulate antiapoptotic proteins, and downregulate proapoptotic proteins expression to protect neuron, reverse age-related changes, and decrease cognitive decline for AD prevention.

In humans, DHA levels do not change in the healthy hippocampus between the ages of 33 to 90 years [53], but, in AD patients, they are reduced by 53% to 8% of total fatty acids in the PE fraction compared to 17% in healthy age-matched controls [53], and, in patients with mild AD, fish oil supplementation (600 mg of EPA + 1700 mg of DHA/day for 6 months) increased plasma DHA and EPA levels, decreased IL-1*β*, IL-6, granulocyte colony-stimulating factor and prostaglandin F_2*α*_ after the stimulation of peripheral blood mononuclear cells with lipopolysaccharide, regulated inflammatory gene expression, and delayed cognitive decline [84–87]. However, in the same study, IL-6, TNF-*α*, A*β*, tau protein, and phosphorylated tau protein in cerebrospinal fluid were not changed in AD patients with fish oil supplementation [88]. In addition, serum DHA levels in AD patients gradually decrease with the severity of clinical dementia compared to healthy age-matched controls [18], while AD risk is reduced, and cognitive decline is delayed by higher DHA levels in blood [89, 90] or *ω*-3 PUFA consumption [91, 92]. These studies indicate that *ω*-3 PUFA consumption reduces AD pathology, inhibits inflammatory cytokine secretion, upregulates anti-apoptotic proteins, and downregulate pro-apoptotic protein expression to protect neuron, reverse age-related inflammatory changes, and decrease cognitive decline for AD prevention summarized in Figure 3. 

## 5. Beneficial Effects of *ω*-3 PUFAs in Cancer: Involvement of Cytokines and Lipid Mediators

### 5.1. Inflammatory Cytokines on the Pathogenesis of Cancer: Modulation by *ω*-3 PUFAs

An impressive body of evidence supports the existence of a relationship between chronic inflammation and the development of some kinds of cancer. Among them cervix, colon, bladder, gastric, liver, esophageal, ovarian, and prostate cancer are included [93–95]. All these cancers have been called “inflammation-related” tumors, since they share the feature to unfold slowly on a background of chronic injury and inflammation. In turn, the definition “cancer-related inflammation” has been also widely used to indicate the link that many epidemiological, experimental and clinical studies have established between persistent inflammatory conditions and the upsurge of cancer in the inflamed tissues of these patients. Altogether, it has been deduced that at least 25% of tumors have a causal relationship with a preexisting inflammation affecting the tissue where the cancer arises [94, 96]. Moreover, in many tumors, such as breast cancer, for which a clear inflammatory pathogenesis has not been directly established, the typical histological and molecular features of a “cancer-related” inflammation are still evident [96]. 

Beside the observations that the “inflammation-related” tumors are rich in inflammatory cells and often surrounded by inflamed tissues, the hypothesis of their inflammatory pathogenesis is also supported by the observation that the incidence of these cancer may be reduced by the administration of anti-inflammatory drugs [97]. Many important mechanistic pathways underlying inflammation associated to cancer have been so far identified, even though the molecular links interconnecting inflammation and cancer are not completely understood [98]. A series of molecular mediators able to trigger inflammation and simultaneously induce oncogenic pathways has been uncovered, including some bioactive eicosanoids originated by the metabolic conversion of AA, as well as reactive oxygen or nitrogen species (ROS and RNS) and cytokines, which are produced by both the inflammatory cells present in the microenvironment surrounding the “inflammation-related” tumors, and by the tumor cells themselves [96, 98, 99]. 

### 5.2. Macrophages and Tumorigenesis

A central pathogenetic role has been attributed also to the polarization of the tumor environment infiltrating macrophages towards the M1 phenotype in preneoplastic environment, then substituted by M2 mononuclear cells in advanced cancer lesions [100]. M1 are high producers of proinflammatory cytokines TNF-*α*, IL-1 and IL-12 and secrete the immunosuppressive IL-10 and transforming growth factor *β* (TGF-*β*) at low levels. The high production of pro-inflammatory TNF-*α*, IL-1 and IL-12 is driven by the activation of the transcription factor NF-*κ*B in these macrophages. On the other hand, M2 macrophages show scarce NF-*κ*B activity that leads to low production of TNF-*α*, IL-1, and IL-12 and high production of immunosuppressive IL-10 and TGF-*β* [100, 101]. 

Among the cytokines that have been recently recognized as important pathogenetic factors in the induction of the growth, invasion, and metastasis of “inflammation-related cancers” the most prevalent and studied in tumors microenvironment are TNF-*α*, IL-6, and IL-1. The detailed description of their pathogenetic role in cancer is beyond the scope of these review, and a recent exhaustive review has been recently published on the subject [96]. The role exerted by the inflammatory cytokines in the development and progression of “inflammation-related” tumors has been further clarified as the resolving phase of acute inflammation and the molecular factors involved in it are being better comprised. It is becoming increasingly clear that the derangement of the processes involved in the resolution of acute inflammation facilitates the onset of chronic inflammation conditions and related cancer [94]. Interestingly, it has been observed that several bioactive lipid mediators endogenously formed and exhibiting a strong proresolving activity play protective roles against tumorigenesis. One hypothesis that has been put forward to explain their effect is that they may inhibit the production of cytokines involved in tumorigenesis [94]. 

### 5.3. *ω*-3 PUFAs and Tumorigenesis

As described above, *ω*-3 PUFAs are known for having strong anti-inflammatory activity. To explain their potent anti-inflammatory action, it has been especially invoked their inhibitory effect on the production of inflammatory eicosanoids derived from AA metabolism, as well as their ability to modulate the production of cytokines and ROS [102]. Recently, their ability to be metabolized to proresolving molecules (i.e., resolvins) and to influence the prevalence of the M1/M2 mononuclear cell phenotype in tissues [20, 103, 104] has been also invoked. We have just considered that all these molecules and factors influenced by *ω*-3 PUFAs have been also recognized as the main mediators involved in the development and progression of “inflammation-related” tumors (see above). On this basis, and in relation to the antineoplastic role of these FAs, supported by over two decades of extensive investigation [105], it is becoming clear that a pathway through which they could exert their anti-cancer action against “inflammation-related tumors” could be the modulation of the activity of inflammatory cells and factors found in the context of these tumors. In this section of the review we focus on the recent research showing a clear relationship between the anti-inflammatory and antineoplastic effects of *ω*-3 PUFAs. We pay particular attention to those findings that support the *ω*-3 PUFA modulating effect on cytokine production in inflamed tissues as a crucial mechanism underlying the antineoplastic effect of these dietary compounds. So far, actually, this issue has been barely studied in a direct way, and only a few published reports are available at the moment, even though it should be underlined that the role of *ω*-3 PUFAs, and especially for EPA, as anticachectic agents reducing the cytokines production in patients bearing advanced tumors is already well recognized [106–108]. For instance, one interesting and recent work by Lou et al. [109] demonstrated that a *ω*-3 PUFA-rich diet had beneficial effect against UVB-induced carcinogenesis. Besides reducing incidence and size of different skin tumors and increasing the latency for their development, *ω*-3 PUFA treatment markedly decreased the levels of several inflammatory cytokines in UVB-treated epidermis, including lipopolysaccharide-induced CXC chemokine (LIX), soluble tumor necrosis factor-alpha receptor 1 (sTNF R1), and macrophage inflammatory protein-1c (MIP-1c). Many other cytokines, already scarcely present in control rat epidermis, were not any more observable in the epidermis of *ω*-3 PUFA-treated rats. In agreement, *ω*-3 PUFAs were found to inhibit *in vitro* the expression of IL-1*β* and IL-6 cytokines in AR42J pancreas acinar tumor cells stimulated by the pancreatitis-inducer cerulean [110]. Moreover, recently Rosa et al. [111] found that in rats treated with fish oil (FO) (Galena FO, 4% w/w) and subjected to 1,2-dimethylhydrazine (DHM)-induced colon carcinogenesis a lower incidence of aberrant crypt foci (ACF) was observed as compared to soybean-oil-treated rats (controls), confirming a wide series of previous observation [112]. Interestingly, the authors suggested the existence of a relationship between the anticancer effect of *ω*-3 PUFAs and their anti-inflammatory effect, since they observed an increased expression of the immunosuppressive factor TGF-*β* and a decreased expression of the pro-inflammatory and pro-angiogenic chemokine IL-8 in the colonic mucosa of DHM-treated rats fed with a FO-enriched diet. This result is in agreement with previous findings indicating that *ω*-3 PUFAs inhibit neo-angiogenesis in animal models of inflamed mucosa and colon cancer [113, 114], and with the observed *ω*-3 PUFA-induced inhibition of IL-8 production in IL-1*β*-stimulated endothelial cells (HUVEC) [113] or in UVB- or TNF-*α*-stimulated HaCaT keratinocytes [115]. Moreover, as it is known that both IL-8 and TGF-*β* are among the cytokines secreted at high levels and specifically by the M2 monocytic phenotype [116], this finding suggests that *ω*-3 PUFAs may exert direct influence on the polarization of M1/M2 monocytic cells in the context of tumor microenvironment. In agreement, Chiu et al. [117] recently found that 17-HDHA, a DHA-derived resolvin, increases the macrophage polarization towards the M2 phenotype *in vitro*. However, these *ω*-3 PUFA-induced alterations may be tissue specific, since a dietary treatment with *ω*-3 PUFAs of transgenic LDLr−/− or apoE−/−mice, an atherosclerosis animal model, did not alter the M1/M2 proportion in blood, whereas it induced M1 polarization in spleen [104]. The monocyte alternating between the two phenotypes M1 and M2 in tumor microenvironment is currently a field of great interest [100], but we should underline that its relationship with the development and progression of “inflammation-related cancers” are presently far from being completely understood. In particular, the hypothesis of Bögels et al. [116] that the polarization of M1/M2 monocytes in the context of tumor microenvironment may be greatly influenced by the specific microenvironments of different tumor types should be noted. These authors found that monocytes grown *in vitro* in the presence of breast cancer cell supernatant showed an increased expression of IL-8 and other cytokines (IL-10, and the chemokines CCL17 and CCL22), all associated with the alternatively M2 phenotype [116]. By contrast, colon cancer cell supernatants induced monocytes to produce more pro-inflammatory cytokines (i.e., IL-12 and TNF-*α*) and ROS [116]. 

It has been widely reported that *ω*-3 PUFAs exert inhibitory action towards NF-*κ*B activation [3]. This effect has been largely invoked to explain the anti-inflammatory and antineoplastic effects of these FAs, since NF-*κ*B is the main transcription factor involved in the upregulation of inflammatory cytokines, and other inflammation related genes (such as COX-2 or genes codifying adhesion molecules), as well as cell growth-related genes [118]. Recently, a dynamic model of gradual NF-*κ*B inhibition associated with M2 polarization in monocytes associated with tumor microenvironment was proposed [100]. According to this model, the full activation of NF-*κ*B in inflammatory leukocytes resident in preneoplastic sites may exacerbate local M1 inflammation (with high levels of TNF-*α*, high IL-1, and IL-12 and low levels of IL-10 and TGF*β*) and favour tumorigenesis [119]. On the other hand, tumor growth may be associated with the progressive inhibition of NF-*κ*B and in the progressive development of M2 inflammation, characterized by low levels of TNF-*α*, IL-1, and IL-12, and high levels of IL-10 and TGF-*β* [120]. On these bases, it can be hypothesized that the preventive or therapeutic efficacy of NF-*κ*B suppressor agents like *ω*-3 PUFAs against cancer could be subject to tumor stage and polarization of infiltrating leukocytes. This means that they could exert a beneficial major role preventing the development of cancer, but scarcely or negatively affect the progression of established and advanced cancer lesions. 

A lower activity of NF-*κ*B was recently observed in colon tumors induced in *fat-1* mice by using the DSS/azoxymethane (DSS/AOM) colon tumorigenesis protocol. These transgenic mice, showing an endogenous tissue enrichment with *ω*-3 PUFAs, had a lower tumor load, and also higher expression of TGF-*β* in the colon [121]. Also in another study performed using *fat-1* mice and the same model for tumorigenesis a decreased incidence of colonic adenocarcinomas was shown, as well as increased apoptosis, decreased CD3(+), CD4(+) T helper cells, and *ω*-6 PUFA-derived eicosanoids in colon cancer tissue [122]. Interestingly, the authors found also decreased macrophage cell numbers per colon, which is in keeping with the decreased number of monocytes of both M1 e M2 phenotype infiltrated in the adipose tissue of animal treated with a diet at high level of FO [103]. Subsequently, several other studies demonstrated decreased liver tumorigenesis in *fat-1* mice. In a transgenic hepatoma model containing double mutations in c-myc and TGF-*α* a constitutive increase in *ω*-3 PUFA tissue content led to protection from liver tumor development that was associated with a decrease in NF-*κ*B levels [123]. In the diethylnitrosamine (DEN) model of chemically induced liver carcinogenesis a tumor suppression effect of increased *ω*-3 PUFA tissue status was demonstrated in the *fat-1* mice as well, with lowered plasma TNF-*α* levels and decreased liver COX-2 expression [31]. Consistent with the view that COX-2 represents an inflammation-associated factor involved in the pathogenesis of “inflammation-related cancers” [124], plenty of work has considered the *ω*-3-PUFA-induced reduction of COX-2 expression as essential to explain the antineoplastic effect of these FAs on tumors that are pathogenetically related to chronic inflammation [114, 125, 126]. 

It has also been suggested that inflammatory components of the tumor microenvironment may impact on mechanisms essential for the generation of metastatic variants [127]. The production of inflammatory cytokines such as TNF-*α* and IL-1 by tumor-associated macrophages can act as potent stimulator of metastasis [96, 128]. Kim et al. [129] recently reported that *ω*-3 PUFAs, besides being powerful inhibitors of the production of these cytokines by macrophages, could exert antimetastatic effects by suppressing the invasive and metastatic response of cancer cells to these cytokines [129]. In particular, they observed that EPA inhibited the TNF-*α*-induced expression of a matrix metalloproteinase (MMP-9) specifically associated to tumor cell invasion and metastases [130, 131] in HaCaT keratinocytes. 

It was recently reported that *ω*-3 PUFAs inhibit EGFR signalling in breast cancer cells [132, 133]. This receptor has been considered the “central hub” capable of integrating and transducing a variety of signals that can have an impact on cancer progression, including inflammatory signals from cytokines and other inflammatory mediators, and accumulating observations support the involvement of EGFR signalling dysregulation in hepatocarcinogenesis [95]. Similarly, it was observed [134] that *ω*-3 PUFAs inhibit ovarian cancer cell growth by specifically inducing TGF-*β*1 expression and modulating molecular pathways and get involved in cancer cell growth. Since it is known that this cytokine may exert both pro- and anti-inflammatory actions, depending on the local tissue environment [135], it would be interesting to investigate if the effect of *ω*-3 PUFAs on the expression of this cytokine may be related to an anti-inflammatory action in the contest of the ovarian cancer microenvironment.

Certainly, the inflammatory hypothesis does not exclude that other routes may exist through which *ω*-3 PUFAs may exert their anticancer action. It has been shown that these fatty acids may exert antineoplastic effects also in models of cancers not showing a major inflammatory pathogenesis (i.e., leukemia or breast cancer) [4, 136]. Moreover, most of the mechanisms that have been suggested to explain their antineoplastic effects have been identified using cancer cells cultured *in vitro* [137], that is, using artificial conditions of cell growth that exclude the presence of the inflammatory microenvironment surrounding the tumors *in vivo*. A number of the identified mechanisms through which *ω*-3 PUFAs may act are common to cancer cells originating from different tissues, whereas others can be cell specific [114, 125, 126, 138, 139]. For instance, we found that *ω*-3 PUFAs exerted antineoplastic effects (i.e., proapoptotic and pro-differentiating effects) on colon cancer and melanoma cells by inducing opposite effects on the intracellular location of *β*-catenin [126, 140].

On the whole, however, unifying routes of action have been identified to explain the beneficial effects of these FAs [3], and it has now become clear that, once they are incorporated in our cell membranes, they can chemically and physically alter that microenvironment and, thus, influence the activities of many membrane-bound proteins with carrier, channel, enzyme, or signaling functions [141]. Moreover, as discussed above, they can also be metabolized in the cells and converted to powerful bioactive products able to modulate many cellular process and functions [20]. It is worth stressing that all effects may be relevant on different levels, either if *ω*-3 PUFAs are incorporated by the tumor cells themselves, or also by incorporation into inflammatory/immune cells surrounding the tumors, and—influencing the process of tumorigenesis—by affecting the normal cells from which tumor cells may originate.

### 5.4. Antitumor Evidence in Humans

There is currently some direct evidence for an anti-tumor effect of *ω*-3 PUFAs on human colon and liver. Regarding an antiproliferative action of *ω*-3 PUFAs in the colon, over 20 years ago a pioneer study was conducted by Anti et al. [142], demonstrating that the abnormal proliferation observed in the colonic mucosa of patients at high risk of colon cancer for sporadic polyposis was suppressed by a combined supplementation with EPA and DHA. This study was confirmed later by Huang et al. [143] who observed that the abnormal proliferation of the colonic epithelium adjacent to surgically resected cancer in patients at high risk of developing a second tumor was reduced by supplementing a fish oil concentrate. In agreement, in a more recent trial in patients with familial adenomatous polyposis the treatment with EPA for 6 months was associated with a reduction in polyp number, a decrease in the sum of polyp diameters and stabilization of polyp burden [144]. *ω*-3 PUFAs might thus be a safe chemopreventive agent in this context. Furthermore, a recent Japanese epidemiological study found that the consumption of *ω*-3 PUFA-rich fish and *ω*-3 PUFAs were associated with decreased development of hepatocellular carcinomas [145].

## 6. Conclusions 

Taken together, a large amount of experimental evidence supports the anti-inflammatory and anti-tumor role of *ω*-3 PUFAs, and numerous mechanisms for these effects have been elucidated *in vitro* and *in vivo*. These studies have led to a better understanding of the influence exerted by *ω*-3 PUFAs and their metabolic derivatives on inflammation/resolution, as well as on the molecular pathways underlying the production and action of inflammatory cytokines in the context of inflammatory, metabolic, and neurodegenerative diseases and inflammation-related cancers. However, so far the clinical application of this knowledge has been lagging behind, which is due, at least in part, to inconclusive or contradicting results in a wide variety of clinical studies using *ω*-3 PUFAs. 

The purpose of this review is therefore to refocus on what is known and what is established. For the clinics this is, beyond doubt, the triglyceride-lowering effect of the *ω*-3 PUFAs. 

However, it is our belief, that future human studies will also have to take a better look at the anti-inflammatory, anti-degenerative, and antiproliferative effects of *ω*-3 PUFAs and their lipid mediators. It is conceivable that the heightened public interest in *ω*-3 PUFA (fish oil) fatty acids in recent decades has already led to an increase in the *ω*-3 PUFA uptake in large parts of western populations, leading to a shifting baseline in intervention studies using *ω*-3 PUFA supplementation and thereby confounding a potential outcome advantage of *ω*-3 fatty acid supplementation. This could be addressed by more widespread measurements of *ω*-3 and *ω*-6 fatty acid levels in the context of these studies—a measurement that has been lacking from most *ω*-3 PUFA intervention studies performed so far. 

Much remains to be done, and given the favorable safety profile of long-term *ω*-3 PUFA supplementation we propose that testing long-term preventive effects of *ω*-3 PUFAs in the context of cancer and neurodegenerative disease, as well as in the metabolic syndrome, should become the most important field of *ω*-3 PUFA research in the coming years.
